# Factors Influencing Online Mental Health Forum Use for People from Ethnic Minority Backgrounds in the United Kingdom: A Mixed Methods Study

**DOI:** 10.3390/ijerph22111638

**Published:** 2025-10-28

**Authors:** Connor Heapy, Paul Marshall, Zoe Glossop, Suman Prinjha, Fiona Lobban

**Affiliations:** 1 Spectrum Centre for Mental Health Research, Divison of Health Research, Health Innovation One, Sir John Fisher Drive, Health Innovation Campus, Bailrigg, Lancaster LA1 4AT, UK; p.marshall4@lancaster.ac.uk (P.M.); z.glossop@lancaster.ac.uk (Z.G.); f.lobban@lancaster.ac.uk (F.L.); 2 Department of Health Sciences, Seebohm Rowntree Building, University of York, Heslington, York YO10 5DD, UK; suman.prinjha@york.ac.uk

**Keywords:** mental health forum, forum use, online support, ethnic minority, South Asian

## Abstract

Background: Ethnic minority groups are under-represented in their use of community mental health services in the UK. Online mental health forums could be a more appealing support option than traditional mental health services. Part one of this study investigated the level of online forum use in people from ethnic minority groups. Part two investigated the factors influencing online mental health forum use for people from ethnic minority groups. Methods: Part one involved comparing data from a range of pre-existing national datasets, and datasets local to Berkshire, UK (i.e., on the general population, people experiencing common mental health problems, users of mental health forums, and NHS Talking Therapies services). Part two involved interviewing 14 individuals from ethnic minority backgrounds who had used, or considered using, online mental health forums. Results: In part one, nationally, Asian, Black, and Mixed ethnic groups appeared over-represented in their online mental health forum use based on their reporting of common mental health problems. In Berkshire, people from Asian and Black ethnic groups were under-represented in their use of Berkshire NHS Trust’s online mental health forum based on their representation in the Berkshire population. In Part Two, three themes were identified as influencing forum use: (1) sense of community in the online and offline worlds, (2) trust is crucial, and (3) barriers to accessing online forums. Conclusion: People from ethnic minority groups vary in their use and experiences of mental health forums. Whilst forums can offer a valued accessible space for anonymous sharing of often stigmatised experiences, pathways to access require trusted figures to promote their availability, and forum designers and moderators to co-create culturally sensitive spaces with people from these target communities.

## 1. Introduction

### 1.1. Background

Approximately one in six adults in the United Kingdom (UK) are affected by mental health problems at any time [1]. Mental health problems not only have a significant human cost, causing distress and disability to the individual and their families, but also a substantial economic cost. For example, mental health problems cost the UK economy around £118 billion per year, mostly due to lost productivity and costs incurred supporting informal unpaid carers [2]. Effective treatments are available and the proportion of people receiving mental health support in the UK is increasing; however, most people with mental health difficulties are still not receiving any support [1]. Access to support is also not equitable across all demographic groups.

People from ethnic minority groups are under-represented in their use of National Health Service (NHS) community mental health services, despite being equally, or more likely, to experience common mental health problems than White British people [1]. Ahmad et al. (2021) analysed one of the largest nationally representative datasets available in the UK, and using data from 2014, they found that Asian, Black, and “White Other” ethnic groups were less likely to receive mental health treatment (i.e., medication, or speaking to a GP, psychiatrist, therapist, or mental health nurse) than the White British group, even after controlling for important demographic and clinical variables (adjusted odds ratios ranged from 0.23 to 0.67) [3]. Similar findings were reported in a study looking specifically at data from the national Talking Therapies for Anxiety and Depression services (previously named Improving Access to Psychological Therapies [IAPT]) collected between 2013 and 2016 [4]. Compared to the White British population, Black African, Asian, and Mixed Ethnic groups were less likely to self-refer to four South London-based Talking Therapies services (46% of overall referrals vs. 40.8–43.4%). Further, compared with the White British group, all ethnic minority groups were less likely to receive treatment following referral and assessment (72.4% vs. 66.1–67.5%) [4]. Disparities in rates of mental health problems, and utilisation of mental health services, appear to have worsened in recent years due to the disproportionate impact of the COVID-19 pandemic on particular ethnic minority groups [5,6,7]. Importantly, this pattern of under-utilising mental health services by ethnic minority groups appears reversed for involuntary mental healthcare, where Black and Asian people are significantly over-represented [8].

Many reasons have been proposed for this under-representation of people from ethnic minority groups in receiving support from NHS community-based mental health services. For example, they might receive mental health support through faith centres and community organisations rather than through the NHS. A systematic review and meta-synthesis of five qualitative studies, including 123 ethnic minority participants (mostly from Black or Asian groups), identified four explanatory themes [9]. The first theme was “structural factors” and included lack of awareness of services available, language barriers, and social issues (e.g., financial problems). The second theme was “perception and belief of service users” and included mental health stigma within communities and differences in how mental health problems are conceptualised. The third theme was “stigma due to cultural differences” and included resistance to psychiatric labelling and experiences of racism from healthcare providers (a review also found experiences of racism in mental healthcare were a major barrier to accessing support [10]). The fourth and final theme was “overall cultural barriers” and included a perceived lack of cultural competence within services. Although not explicitly discussed in the review, mistrust of services was a theme identified in some of the included studies [11] and has also been identified as an important barrier in a larger survey-based study [12]. A systematic review of barriers to mental healthcare for African immigrants in the UK identified some similar barriers, including stigma, discrimination, and financial problems [13]. The review also highlighted some additional barriers, including denial of the presence or severity of the problem, and lack of self-confidence and self-efficacy. Overcoming these identified barriers is vital in increasing access to mental health services for ethnic minority groups.

Digital psychological support, such as that offered through online mental health forums, could be a more appealing option for people from ethnic minority groups than more traditional options such as medication or face-to-face therapy. Online mental health forums are spaces where individuals with mental health difficulties can share and discuss mental health information and support. Often set up by healthcare providers and charities, these online spaces facilitate peer-to-peer support and have the potential to overcome some of the barriers to access identified in previous research [9]. First, forums are usually anonymous and can be accessed discretely from anywhere with a device and internet connection [14]. As a result, individuals are less likely to be identified when seeking support, reducing the barriers of stigma or racism [15]. Second, forums can be freely accessed anywhere, reducing financial barriers associated with attending in-person appointments, such as travel, and time [16]. Third, forums may not be perceived as typical healthcare services and may therefore be trusted more than traditional support options such as therapy. Fourth, as those from ethnic minority groups may lack awareness of NHS services for mental health support [9], forums are spaces where people can signpost and share information about these other services. In addition, a systematic review highlighted that forums have the potential to be unhelpful in particular circumstances. For example, where forums do not feel like nonjudgemental spaces, where harmful behaviours (e.g., self-harm) are openly discussed, and where forum moderators are slow to respond or inconsistent in the enforcement of forum-rules [17]. Importantly, these proposed barriers and facilitators to forum use for ethnic minority groups are speculative due to the lack of research evidence in this area.

### 1.2. Objectives

The aim of this study was to investigate levels of online mental health forum use by people from different ethnic groups, and to explore their views about these forums. This study is part of the larger “Improving Peer Online Forums (iPOF) study [18] and is, to the author’s knowledge, the first to attempt to answer the following research questions:

(1) What is the level of online forum use in people from ethnic minority groups? (Part one)
(2) What are the factors influencing online mental health forum use for people from ethnic minority groups? (Part two)

## 2. Methods

### 2.1. Part One

#### Participants and Procedure

To assess whether people from diverse ethnic minority groups are under-represented in their use of online mental health forums, the proportion of each ethnic group (i.e., the number of people from each ethnic group, divided by the total number of people, multiplied by 100) was presented and compared descriptively using the aggregate, non-identifiable UK national datasets listed below. Inferential statistics were deemed inappropriate for this data due to not meeting the assumptions of non-parametric tests. More specifically, the groups may not be independent and numbers for some ethnic groups are low. In addition, ethnicity data are not captured in a uniform way across forums and there is a significant amount of missing data, though it is not clear whether this is missing at random. Data from several online mental health forums have been used in this study. These forums were selected as part of the broader iPOF study [18]. Pseudonyms have been created for each of the forums to protect the identity of users. A summary of each forum is available here: https://www.lancaster.ac.uk/ipof/case-summaries/ (accessed on 16 October 2025).

- The general population taken from the Adult Psychiatric Morbidity Survey (data from 2024 [1]).
- People with common mental health problem taken from the Adult Psychiatric Morbidity Survey (data from 2024 [1]). Common mental health problems were defined in this survey as depression and anxiety disorders.
- People with bipolar disorder taken from the Adult Psychiatric Morbidity Survey (data from 2024 from [1]).
- People who attended at least one treatment session within Talking Therapies national services, taken from NHS Digital [19].
- People who had used a Bipolar specific online forum (Robin) within the six months prior to January/February 2022. These people were experiencing bipolar themselves or have a family member/friend with bipolar disorder, and had opted to complete an online survey (i.e., this does not represent all users of the forum).
- People who had used an online forum hosted by a charity service for young people (Chaffinch), within six months prior to September–December 2022. These people had opted to complete an online survey (i.e., this does not represent all users of the forum).
- U.K members of an online mental health and wellbeing service including a forum (Jay); data from 2022.

The proportion of each ethnic group using different NHS mental health services within one geographical area within the UK were also presented and compared descriptively. The county of Berkshire was selected as the Berkshire NHS Foundation Trust offer a range of community mental health services, and they were also one of the first Trusts to offer an NHS online forum as part of an NHS mental health service. The datasets examined were as follows:

- Berkshire census data [20].
- People who have attended at least one appointment in the following mental health services in Berkshire NHS Trust: adult community mental health teams, adult eating disorders, perinatal, child and adolescent eating disorders, and child and adolescent anxiety and depression (2022–2023).
- People who have entered treatment with Berkshire Talking Therapies services (in 2022–2023).
- People using the Support Hope and Recovery/Resources *Online* Network (SHaRON; online mental health forum run by Berkshire NHS Trust—all users registered up until the end of 2021).

### 2.2. Part Two

#### 2.2.1. Participants and Procedure

A qualitative individual interview study was conducted to understand forum use in people from ethnic minority groups. In total, 14 participants were recruited to this part of the study. Participants were recruited from a range of sources including the iPOF study’s patient and public involvement (PPI) group, university volunteer lists, third sector organisations, social media, online mental health forums, and snowballing (i.e., asking participants to share the study details with eligible friends). The advert invited participants to take part if they met the following inclusion criteria:

- Currently living in the UK;
- Identified as being from an ethnic minority group (i.e., not White British);
- Self-identified as having previously experienced mental health struggles;
- Had either used, or considered using, online mental health forums.

Interested participants first completed an online consent form and short self-report demographic questionnaire which assessed age, gender, and ethnicity. Participants were then purposively selected for interview to ensure variation in age, gender, ethnicities, and forum experience. Participants were selected for interview on an ongoing basis, as they expressed interest. All selected participants were interviewed by the lead author either by telephone or using Microsoft Teams (either with or without the camera function enabled). Interviews were semi-structured and ended when participants had nothing further to say in response to the questions (duration was between 20 and 55 min). The interview guide was developed through discussion within the research team, in line with the study’s objectives, and assessed participants’ knowledge and awareness of forums, as well as barriers and facilitators of use (see Supplementary File S2 for topic guide). Participants were compensated with a £30 gift voucher for their time. Recruitment stopped after 14 interviews due to time constraints within the study. Ethical approval was granted for both parts of the study by NHS Solihull Research Ethics Committee, UK (reference: 22/WM/0132).

#### 2.2.2. Analysis

Interviews were audio-recorded by the interviewer and transcribed by a professional transcriber. Interviews were then analysed in NVivo (v.12) by following the six steps of reflexive thematic analysis [21]. This process involved the lead author first reading and re-reading the transcripts for familiarisation, making brief notes on any analytic ideas that were generated. Next, the lead author worked systematically through the dataset of transcripts and coded sections of data, using both semantic (i.e., explicit, surface level) and latent (i.e., conceptual or implicit meaning) codes, based on the main research question. Following this, the lead author arranged codes into initial candidate themes based on similarities in meaning. All relevant coded data was collated into each candidate theme. Candidate themes were then reviewed and checked against coded data and the research question in an iterative process. Some candidate themes were discarded and others collapsed together. Themes were also discussed within the research team before final themes were generated, theme names finalised, and a brief description of each theme was written up. Finally, detailed descriptions of the themes were written up to create an analytic narrative relevant to the research question.

#### 2.2.3. Reflexivity

The lead author who conducted the interviews was a White British cis-gender male who was keen to develop skills to understand participants’ experiences in a culturally sensitive way. Participants may not have felt comfortable sharing sensitive cultural or personal information with the interviewer; or the interviewer may have inadvertently created an awkward conversation in their attempts to be engaging. Following advice from two members of a Patient and Public Involvement (PPI) group who identified as South Asian, the interviewer acknowledged their ethnicity and potential lack of cultural awareness at the start of each interview. In addition, the interviewer asked participants what term they preferred to be used when referring to their ethnicity. The interviewer also maintained a reflexive journal throughout the interview process to increase self-awareness and enhance transparency in reporting the analysis. Of note, these interviews were conducted shortly after the COVID-19 pandemic and in the wake of media reporting of inequalities in access and effectiveness of vaccines within ethnic minority groups, which may have been relevant here.

Three other authors also identified as White British. FL and ZG are cis-gender females, and PM is a cis-gender male, each with an interest in increasing access to support for mental health. SP is a cis-gender South Asian female of Indian heritage, with an interest in ethnic health inequalities. She is also a BACP-registered psychotherapist. Authors engaged in reflexive discussion and provided feedback on the analysis to promote analytic rigour.

## 3. Results

### 3.1. Part One

See Table 1 and Table 2 for the number, and percentage proportion of, people from different ethnic minority groups across national and local forums, national and local population cohorts, and national and local mental health service use. To summarise some key findings from the national data (Table 1):

Asian/Asian British individuals reported common mental health problems less than expected (5.8%) based on their representation in the UK population (7.2%). Asian/Asian British individuals are over-represented in having attended at least one treatment session in Talking Therapies services (7.4%), in their use of the Chaffinch (6.4%), and under-represented in their use of Jay forums (5.3%), based on the number reporting common mental health problems (5.8%).

Fewer Black/Black British individuals reported common mental health problems than would be expected (3.1%) based on their representation in the U.K population (4.1%). They attended at least one treatment session in Talking Therapies services more than expected (4.4%) based on the number reporting common mental health problems (3.1%). Black/Black-British individuals were over-represented in their use of the Chaffinch forum (10.6%) based on the number reporting common mental health problems (4.1%). They were also over-represented in reporting bipolar disorder (5.9%) and were under-represented in their use of the Robin forum (0.7%). Data on Black/Black British individuals use of the Jay forum was unavailable.

White British individuals reported common mental health problems (83%) and bipolar disorder (83.8%) more than expected based on their representation in the U.K population (80.2%). White British individuals attended at least one treatment session in Talking Therapies services about as much as expected (82.2%) based on the number reporting common mental health problems (80.2%). They were under-represented in their use of the Chaffinch (72.3%) and Jay (64.9%) forums, but over-represented in the Robin forum (89%), based on the number reporting common mental health problems (83%).

To summarise some key findings from the local data:

Asian/Asian British individuals appear under-represented in using Berkshire mental health services (12.0%) based on their representation in Berkshire (17.1%). They were also under-represented in receiving at least one treatment session from Berkshire Talking Therapies services (12.7%), and in their use of the online forum, SHaRON (6.7%).

Black/Black British individuals appear slightly over-represented in their use of Berkshire mental health services (4.7%), and in receiving at least one treatment session from Berkshire Talking Therapies services (4.3%), based on their representation in Berkshire (3.8%). They appear under-represented in their use of the online forum, SHaRON (1.7%).

White British/Other individuals appear over-represented in using Berkshire mental health services (76.3%), Berkshire Talking Therapies Services (77%), and in their use of the online forum, SHaRON (85.3%). based on their representation in Berkshire (73.1%). 

### 3.2. Part Two

See Table 3 for participant information. Most participants identified as South Asian/South Asian British (11/14). No participants identified as Black/Black British. Most participants (9/14) had experience of using online mental health forums. Most participants (9/14) identified as female.

Three themes were developed from the data. The first theme explores the need for people to belong to a community in which they feel they can talk openly about mental health challenges, be understood, and be accepted. The second theme highlights the importance of trust in determining how participants initially accessed and subsequently behaved within forums, and the extent to which they were able to benefit from, or offer support to others within the forum. The third and final theme highlights barriers to access, which have clear implications for practice (see Table S1 for further supporting extracts for each theme).

#### 3.2.1. Sense of Community in the Online and Offline Worlds

Feeling part of an online group in which it was possible to talk openly about mental health and where people “can lean on and support each other” (P4) was important, and a key driver for forum use. Online forums offer a way to access mental health support for people living in communities in which mental health is highly stigmatised. For example, some participants described how talking about mental health difficulties in their own communities could be perceived negatively, as a lack of gratitude towards God or grandparents who had worked hard to “make a new life in a different country” (P9). South Asian/South Asian British as well as East Asian/East Asian British participants described rigid expectations about life paths from within their communities. When people deviated from this path, such as by having a mental health difficulty, this could have a negative impact on the family reputation where “if one person has a problem, the whole family looks bad” (P9) and there was a risk of losing community support.

“*Coming from South Asian communities there’s always challenges, for example how the community have this—the South Asian community in particular, they have a good way of coming across like, ‘This is how the community are, this is how we’re perceived, this is how we behave.’ Anything you deemed inappropriate or not considered the norm is a threat to people’s lives, like if you’re excluded you don’t feel counted in so it’s like fitting these labels, these boxes for fitting into the community because any areas of concern or debate like mental health is not a concept the community are ready or able to talk about openly*”.

(P8)

Mental health stigma was generally seen as hindering conversations about mental health in offline communities. This lack of conversation meant that some people within ethnic minority communities “do not understand what mental health is” (P8), and others avoided talking about mental health because “they feel they’ll be judged” (P6). Online mental health forums were therefore a welcome support option for some participants as they felt forum users understood their difficulties better than friends and family. In this sense, online forums served as chosen families/communities, providing a space where participants could form supportive bonds with people who understood them in ways their biological family or offline community could not. One participant who had not used online mental health forums explained that this was because they “had quite a good network of friends, family that have been quite supportive” (P5), highlighting how forums can serve as an alternative online community for support when this is absent in real-world communities. Participants who found forums helpful may therefore have experienced a sense of dissonance from suppressing, or avoiding talking about, parts of themselves when offline that received validation and support online.

Interestingly, the perception that stigma is what prevents people from ethnic minority groups talking about mental health, and engaging with mental health services, was not shared by everyone. One South Asian/South Asian British participant described how they believed that the under-representation of people from ethnic minority groups in mental health services, including online forums, was due to strong community connections that meant that mental health services were not needed. Strong community connections outside of the forums could reduce loneliness and increase a sense support from others.

“*Yeah, don’t assume that because someone is part of a racial minority that they are cut off. We are fully integrated in society. We are fully a part of a society. It’s deeply stigmatising this attitude around if you are this then you are vulnerable to this. That’s not the case. there’s a lot more community support on the ground, put it that way and a lot of more family support as well*”.

(P11)

This comment demonstrates a clear desire to counter stereotypes about particular ethnic groups that the participant feels are unhelpful, including those discussed in the interview.

For another participant, the high levels of stigma within the offline community served to reduce rather than increase online sharing because of fear of being “identified by friend and family on the site” (p14), even within anonymous forums. This risk could potentially be greater in people from minority groups who chose to talk openly about themselves and their lives on the forums. This could create a dilemma for people who feel the wider context of their life is important for understanding their mental distress.

For participants who did use forums, there was a level of understanding from other forum users with shared experiences that was not easily found in offline communities.

“*Family was harder to get them to understand me, whereas [Forum name] was more. you don’t need to go into a big explanation or anything. they have experience with something so they know…*”.

(P6)

However, lack of diversity (e.g., in ethnicity, sexuality) within forum membership was identified as a barrier to feeling part of the community and resulted in participants at times feeling like outsiders and unwelcome in the online space.

“*I guess the thing with [Forum name] as well predominantly cis white males who use [Forum name] so some of the stuff on [Forum name] isn’t the best to be looking at especially if you’re having mental health crisis episodes.*”

(P7)

#### 3.2.2. Trust Is Crucial

Participants described how being recommended forums from people they trusted increased the likelihood that they would access them. Trust was related to how familiar or credible the source was to participants. Religious centres (e.g., mosques) appeared to be particularly trusted by South Asian/South Asian British participants—reflecting the importance of religious institutions—and the perception that “a lot of stuff to do with mental health in South Asian communities is also to with religion” (P7). Doctors were also described by participants as trusted figures, whose advice would be followed. However, some other roles within the healthcare system (e.g., nurse) were given less credibility, and even viewed with some suspicion, suggesting that careful thought needs to be given to how, and by whom, individuals are signposted to different kinds of mental health support, including online forums.

*“…there’s more mistrust because mistrust issues with authoritative figures or in the past people have not shared information correctly with the vaccines or things like that, pharmaceutical companies doing all sorts of things and so it’s a bit of mistrust in things so like GPs, you would trust a doctor but you would not trust a nurse or another health professional that you think oh they are not a doctor so there is this idea that only the doctor is going to be most helpful rather than going to other professional roles just a health practitioner or a link worker or something like that so you tend to give more credibility to the doctor*.”

(P2)

Whilst trust can therefore come from multiple sources, there appeared to be more doubt around mainstream health sources compared to religious institutions embedded within participants communities.

Within forums, participants were more likely to listen to, and value, the advice of others if they trusted them. When other forum users shared personal experiences, whether relating to mental health and/or cultural factors, this appeared to instil trust in the information being shared and increase the likelihood that participants would find the forum posts helpful. Posts from those who shared experiences felt more authentic and relevant to the participants’ situation.

“*Yeah I think you feel less alone and then you also get some sort of real evidence of people navigating what you are trying to navigate through. I think that’s why I kind of turned to that because just a standard Google search would give me a web site like or how to help with depression but it’s so generic. When sharing other people’s stories for me it’s a bit more convincing and a bit more helpful and valuable… I think there’s a slightly different nuance to that in Asian cultures so my questions are around those sorts of things and it’s particularly helpful to get an understanding of the experiences of an Asian person going through that because it is slightly different I think*.”

(P9)

When it came to posting on forums, participants commented on the need to feel they were in a safe and supportive space.

“*I also think if you’re providing a forum for people—if the aim of the forum is to encourage people to open up about their experiences and to help one another with sharing best practice or sharing resources and advice, then I think you need a safe space to allow that to happen or I think people would shy away from that or won’t use it*.”

(P5)

A sense of safety was generated by supportive, non-judgemental responses from others and confidence that personal information would not be shared with people outside the forum. Some participants described fears of their data being leaked from the forum to the outside world, and felt the “online world is not very safe” (P2). Anonymity within forums created a sense of safety for participants who feared that people they knew personally would find out about their difficulties. Anonymity helped them feel “free in expressing what you are feeling” (P13). However, other participants described how anonymity within forums reduced their sense of safety as they were concerned that other forum users would be more critical when their identities were hidden, or because they felt they needed to understand the identity of another person in order to feel comfortable disclosing personal information. Participants who prefer to know more about other’s identities before sharing personal information may opt to use other social media networks, such as Facebook, rather than online mental health forums, where people have profiles containing a photograph and other personal information (as with this participant):

“*I would not be able to share my story if there is no name… That’s me but everyone’s different. People can share their stories without other people knowing who they are but if somebody asked me—if I had joined a forum and I’m happy to share my story, I might as well share my picture, share my name, share my ethnicity and I would feel more comfortable but everybody is different but that’s what I am because I can’t share my personal story with anybody anonymously and I don’t want other people expecting they do the same because I think it is identity is very important*.”

(P1)

Overall, participants who withheld information about themselves to protect anonymity may have created a barrier to feeling truly connected to others, creating a paradox where anonymity both protected them but also prevented them from feeling genuine connection with others.

Moderators provided a key role in making participants feel safe on forums by enforcing forum rules, including the moderation of racist or discriminatory language. One participant described how “if there’s moderators, if there is any kind of comment from a user which is harsh or which is rude, they always take appropriate action, so you feel that you are in a safe space” (P2). Moderators who were perceived to not enforce rules fairly or who were overly punitive (e.g., banning people without warning), were perceived negatively and this sometimes led to participants no longer using online forums. One participant described how they felt some moderators punished people based on a personal dislike of them, rather than a rule violation.

“…*the moderators are—everyone compares them to little dictators. They’re the little dictators of their own… kingdoms … the mods have complete control over what gets posted, what comments and whatever, and so they can act very dictatorial and there are mods out there who are really not great mods and they’ll remove things because they just don’t like you. It has nothing to do with whether or not it meets the rules of the forum or anything like that*.”

(P10)

The knowledge and skillset of forum moderators was also important in making forum users feel safe. Moderators who were qualified healthcare professionals appeared to be particularly trusted by some participants, due in part to the fact that they were seen as working within a broader ethical framework as part of their training.

“*Yeah I would say healthcare professionals are probably better placed just because… they have a clear ethical standard, ethical obligations that… I would think dictate what should and shouldn’t be moderated, what is and isn’t helpful*.”

(P9)

#### 3.2.3. Barriers to Accessing Online Forums

Participants described the accessibility benefits of online mental health forums: “It just feels easy, accessible and it’s free, there’s no waiting list or waiting time for it” (P6). In addition, forums are accessible when alternative methods of support are not available (e.g., during the night).

“*I think it would have been late at night so I guess that was my only support at the time because normally when I do post late at night there’s no one up and I don’t really want to phone a crisis line or anything*.”

(P7)

However, in other ways, forums were experienced as difficult to access. Technology was considered as a barrier to some participants, particularly for older adults either because they “don’t really know about technology” (P4) and were not competent using a device (e.g., phone/laptop) or because it was an unfamiliar form of interaction that did not involve human connection. Second, most forum content is in text form and in the English language, which for many people is not their first language; this created challenges in understanding and expressing complex mental health related experiences. Language was a barrier not only in engaging with the content within the forum, but also in understanding how to navigate the online platform.

“*Yeah, I think it would be a language barrier. They may not speak fluently in English and that may be difficult to access on forums or they may not understand how it would work*…”

(P14)

Finally, some of the mental health language often used on forums e.g., relating to diagnoses, or even just the broad concept of mental health, was perceived by some as stigmatising and a barrier to access. For example, one participant described how “I’ve never ever come across any brother or any Muslim brother who has actually said to me, ‘Our mental health,’” (P8) and that this was, in part, because it is associated with “a word that is used a lot which is ‘madman’ which isn’t useful or helpful.” (P9). Potential users may have therefore avoided forums due to associated stigma or because they did not associate the language with their own experiences.

“*I think something instead of using mental health just use some other word because when someone says like mental health in my country… things like mental health means you are psychological, you are not normal so something—I would suggest using another word besides them*.”

(P3)

This quote highlights the de-stigmatizing potential of alternative, non-pathologizing language. Barriers to accessing forums could therefore be reduced by using language less influenced by a westernized, medical view of mental health, and more influenced by participants specific cultural understanding of mental health.

## 4. Discussion

This was the first study to assess levels of online forum use in people from ethnic minority groups (Part One) and to explore the factors influencing online mental health forum use for people from ethnic minority groups (mostly South Asian/South Asian British; Part Two). The findings from Part One of the study were mixed and therefore difficult to interpret; however, some patterns did emerge from the data. Nationally, Asian, Black, and Mixed ethnic groups appeared over-represented in their forum use based on their reporting of common mental health problems. However, their use of online forums was generally lower than their use of traditional NHS mental health services designed to treat common mental health problems. In Berkshire, data on common mental health problems were unavailable but people from Asian and Black ethnic groups were under-represented in their use of Berkshire NHS Trust’s online mental health forum based on their representation in the Berkshire population, and based on their use of local mental health services for mental health problems. These findings highlight the variation in forum use both between and within different ethnic minority groups in the U.K. One possible explanation for this difference in finding between the national and local datasets is that individuals from ethnic minority group in Berkshire had easier access to traditional mental health services than the average individual from an ethnic minority group in the U.K. The reason for this greater access could be due to the socioeconomic status of Berkshire being higher than the U.K average (as per the Index of Multiple Deprivation ranking [22]), which is associated with greater access to mental health services and more trust in those services that are available [23,24,25].

In Part Two of this study, three key themes were identified as influencing online mental health forum use for people from ethnic minority groups. The first theme highlighted how participants sought a community to discuss their mental health struggles with, and online communities were often deemed as more supportive than real-world communities for this; in part due to stigma experienced within their own communities. The second theme highlighted the importance of trusting others on the forums, from accessing forums, to reading forum posts, to posting on forums. In some cases, trust was generated automatically due to a person’s role (e.g., a religious figure); in other cases, trust was developed when participants felt that they could relate to the experiences or personal characteristics of others, and they were confident that their information would not be accessed by people they knew outside of the forum. The third theme highlighted the accessibility benefits (e.g., available 24 h a day) that motivated some participants to use forums, but also accessibility barriers that participants believed may have prevented others from using the forums (e.g., language, and unfamiliarity with technology).

Together, these themes are consistent with findings from previous studies on barriers to traditional mental health service use in ethnic minority groups [9,11,12,13]. More specifically, inability to speak fluent English, stigma from within communities, and non-identification with medicalized mental health language were highlighted as barriers to accessing traditional mental health services and online mental health forums [9,11,12,13]. However, some differences were also noted in this study. One key difference was that whilst there was some evidence of distrust in healthcare services in this study, linking mental health forums to these services—such as by being recommended by a GP, or moderated by a mental health professional—appeared to increase some participants trust in online mental health forums. This difference may be explained by socioeconomic status, with participants in this study possibly being of higher socioeconomic status than those in previous literature based on their use of technology. Indeed, lower socioeconomic status has been associated with lower trust in healthcare institutions [23,24]. Another finding from this study that appeared to contradict previous studies is that whilst stigma from within communities was a barrier to some participants in accessing online mental health forums (due to fear of being identified), for others, it motivated them toward using forums due to the validation and understanding they received in those spaces. This difference may be due to the anonymous nature of most forums which means users feel more comfortable discussing their experiences than in-person and therefore provides an alternative outlet for support [17].

This study had several strengths—it captured the voices of people historically underserved by NHS mental health services and attempted to triangulate quantitative and qualitative data to inform practical solutions to improve the services offered to these groups. However, this study also had several weaknesses that should be considered alongside the findings. In Part One of the study, the data quality was generally poor with small samples and either large amounts of missing data or a lack of information around missing data. Many different mental health difficulties that may be experienced by those from ethnic minority groups (e.g., trauma, somatization) were also not captured in the data used. In addition, comparisons across datasets were problematic as data were often collected from different years, and there was a lack of consistency and clarity around how people had been grouped based on ethnicity. Finally for Part One, much of the forum data were collected via self-report questionnaire, meaning it was unlikely to be an accurate representation of forum users. For Part Two of the study, the diversity of views collected was limited as most participants described themselves as South Asian/South Asian British and under 55 years old. This skew in sample may be reflective of the recruitment strategy used (e.g., “snowballing” may have lead to a more homogenous group; the third sector organisations used may have had larger networks of younger, South Asian/South Asian British participants). Participants were also likely already familiar with technology as many were recruited online and many interviews were conducted via video conference software. Finally, a variety of remote methods were used in this study (i.e., telephone, Microsoft Teams both with and without the camera enabled) to interview participants and this may also have affected the quality of the data. More specifically, there may have been differences in the data collected from the telephone versus a video call on Microsoft Teams (e.g., verbal cues may be missed, or participants may have felt more comfortable opening up using one format over the other). However, the decision to use a variety of remote methods was chosen in an attempt to increase accessibility and therefore increase the diversity of included participants.

The findings of this study have several important implications for clinicians and service providers. First, raising awareness of mental health forums within ethnic minority groups would likely increase their use. Doctors and community/religious leaders may be particularly well-placed to help promote forums within Asian/Asian British communities, as they are perceived as trusted figures. Second, non-medical and non-pathologizing mental health language should be used when promoting forums and within forums themselves. This may increase the likelihood that people from ethnic minority groups can relate to the purpose of the forum, and reduce the likelihood that they will avoid the forum due to perceived stigma. Third, offering training on how to use the forums could increase engagement. Targeting such training at older adults in particular could help ensure equal access to forums across the lifespan. Fourth, it is vital that online forums demonstrate cultural competence within forums. This could be achieved in many ways including offering forums in which a range of languages are used, providing culturally specific mental health resources, recognising a range of religious holidays within the forums and related communication from forum providers, providing female only spaces to talk, and recruiting moderators from a range of ethnic and cultural backgrounds. Importantly, all of these suggestions should be achieved alongside ethnically diverse experts-by-experience.

The findings of this study highlight some future directions for research. A study using a “think aloud” method [26] could provide useful insights into what makes a forum useable and inclusive. More specifically, participants from a range of ethnic groups could be presented with pre-existing forums and asked to navigate the forum, read responses, and make a forum post, whilst voicing their thoughts. Such a study could provide more specific and fine-grained data about online mental health forums than the current study, whilst also controlling for differences in forum use. Future studies could also explore other characteristics which may be associated with under-representation in NHS mental health services, such as gender, socioeconomic status, and sexual orientation, and how such characteristics intersect in determining forum use. Finally, future research should build upon the comments from one participant that suggested stereotypes around ethnic minority groups are being propagated through research. This participant reported that many people within ethnic minority groups do not experience stigma around mental health, or barriers to accessing mental health services, and are well supported within their communities. Such people may be less likely to take part in research. Future studies should therefore explore ways of improving sampling for research to include a diversity of voices, such as by using the National Institute for Health and Care Research (NIHR) toolkit on increasing diversity in research participants [27].

The findings of this study also point to some future directions around how participant data is collected and categorised. Both mental health forums and NHS mental health services would benefit from accurately capturing ethnicity data. Indeed, this problem has been widely recognised elsewhere [19]. From the current study, it was not possible to conclude with confidence whether people from ethnic minority groups were under-represented in their online mental health forum use, in part due to missing or inconsistent data collected. Forums could ask users to consent to providing basic demographic information when they sign up to the forum. Whilst this may cause some concerns for participants around data security, it will likely be outweighed by the benefits of collecting such data for minoritized groups, such as allowing forums to target under-served groups or allowing them to recruit moderators from similar demographic backgrounds.

## 5. Conclusions

This mixed methods study found people from ethnic minority groups vary in their use of mental health forums, with national data suggesting they are over-represented in their forum use and local data (to Berkshire) suggesting they are under-represented in their forum use. This study also found that whilst experiences varied, some people from ethnic minority groups (mostly South Asian/South Asian British) found online forums as helpful alternative communities in which they could speak about their mental health difficulties without fear of stigma or judgement. Forums were also considered as more accessible support options than traditional mental health services. Trust was a key influencing factor of forum use, with forum use being more likely if participants were confident their data was secure; if participants felt the forum provided a safe and supportive space; and if forums were promoted by a trusted figure. Whilst the picture presented in this study is fairly complex, it provides a valuable foundation upon which future research can build.

## Figures and Tables

**Table 1:** Number and percentage proportion of ethnic groups in national cohorts and online mental health forums.

	General Population ^1^	People Reporting Common Mental Health Problems ^1^	People Reporting Bipolar Disorder ^2^	People Using National Talking Therapies Services ^3^	People Using the Robin Forum ^4^	People Using the Chaffinch Forum ^5^	People Using the Jay Forum ^6^
Asian/ Asian British	497 (7.2%)	85 (5.8%)	4 (2.9%)	83,174 (7.4%)	9 (3.1%)	6 (6.4%)	12,525 (5.3%)
Black/ African/ Caribbean/Black British	279 (4.1%)	45 (3.1%)	8 (5.9%)	49,832 (4.4%)	2 (0.7%)	10 (10.6%)	n/a
Mixed/ Multiple ethnic groups	183 (2.7%)	43 (3%)	2 (1.5%)	39,254 (3.5%)	16 (5.5%)	3 (3.2%)	n/a
White British	5517 (80.2%)	1208 (83%)	114 (83.8%)	926,562 (82.2%)	258 (89.0%)	68 (72.3%)	152,811 (64.9%)
White (Other)	406 (5.9%)	75 (5.1%)	8 (5.9%)	n/a	n/a	6 (6.4%)	25,051 (10.6%)
Other ethnic group	n/a	n/a	n/a	28,438 (2.5%)	5 (1.7%)	1 (1.1%)	47,597 (20.2%)
Total	6882	1456	136	1,127,260	290	94	235,479

Note. Where ethnicity figures do not equal the total, this is a consequence of not having access to original datasets and instead making calculations based on rounded figures from secondary data sources; not specified or missing data was excluded from this table. n/a = data was not available for this specific ethnic group. ^1^ Taken from the Adult Psychiatric Morbidity Survey (data from 2024 [1]). ^2^ People with bipolar disorder taken from the Adult Psychiatric Morbidity Survey (data from 2024 [1]). ^3^ People who attended at least one treatment session within Talking Therapies national services (2022–2023). ^4^ People who had used the Robin forum within the six months prior to January/February 2022. These people were experiencing bipolar themselves or have a family member/friend with bipolar disorder, and had opted to complete an online survey (i.e., this does not represent all users of the forum). ^5^ People who had used Chaffinch online discussion boards within six months prior to September-December 2022. These people had opted to complete an online survey (i.e., this does not represent all users of the forum). ^6^ U.K members of the Jay (an online mental health and wellbeing service; data from 2022).

**Table 2:** Number and percentage proportion of ethnic groups in Berkshire and using Berkshire mental health services (including an online mental health forum).

	Population Covered by Berkshire NHS Trust ^1^	Users of Berkshire NHS Trust Mental Health Services ^2^	Users of Talking Therapies Services in Berkshire ^3^	Users of SHaRON (Run by Berkshire NHS Trust) ^4^
Asian/Asian British	162,743 (17.1%)	790 (12.0%)	2039 (12.7%)	123 (6.7%)
Black/Black-British	36,211 (3.8%)	306 (4.7%)	698 (4.3%)	31 (1.7%)
Mixed/Multi-Ethnic	33,785 (3.6%)	308 (4.7%)	593 (3.7%)	88 (4.8%)
White British	694,075 (73.1%) *	4500 (68.4%)	11,033 (68.8%)	1447 (78.5%)
White Other	n/a	517 (7.9%)	1318 (8.2%)	125 (6.8%)
Other ethnic group	22,960 (2.4%)	155 (2.4%)	367 (2.3%)	30 (1.6%)
Total	949,774	6576	16,048	1844

Note. Where ethnicity figures do not equal the total, this is a consequence of not having access to original datasets and instead making calculations based on rounded figures from secondary data sources; Not specified or missing data was excluded from this table. SHaRON = Support Hope and Recovery/Resources *Online* Network. * Categorised as “White” so includes “White Other”. ^1^ Berkshire census data (2021). ^2^ People who have attended at least one appointment in the following mental health services in Berkshire NHS Trust: adult community mental health teams, adult eating disorders, perinatal, child and adolescent eating disorders, and child and adolescent anxiety and depression (2022–2023). ^3^ People who have entered treatment with Berkshire Talking Therapies services (2022–2023) ^4^ People using the Support Hope and Recovery/Resources *Online* Network (SHaRON; online mental health forum run by Berkshire NHS Trust—all registered users up until the end of 2021).

**Table 3:** Participants demographic data.

Particpants	Age	Gender	Ethnicity	Source of Recruitment	Mental Health Forum User?
1	46–55	Female	South Asian/South Asian British	Patient and public involvement group	No—but has used social media for mental health support
2	36–45	Female	South Asian/South Asian British	Patient and public involvement group	No—but has used social media for mental health support
3	36–45	Female	South Asian/South Asian British	Snowballing (i.e., via another participant)	No—but has used social media for mental health support
4	26–35	Female	South Asian/South Asian British	Social media	No—but has used physical health forums
5	26–35	Male	East Asian/East Asian British	University research volunteer list	No
6	26–35	Male	South Asian/South Asian British	Social media	Yes
7	26–35	Non-binary	South Asian/South Asian British	Mental health forum	Yes
8	16–25	Male	South Asian/South Asian British	University research volunteer list	Yes
9	26–35	Female	East Asian/East Asian British	Third sector organisation	Yes
10	16–25	Female	South Asian/South Asian British	Through a previous related study	Yes
11	26–35	Female	Mixed/Multiple Ethnic Groups	Third sector organisation	Yes
12	46–55	Female	South Asian/South Asian British	Third sector organisation	Yes
13	16–25	Female	South Asian/South Asian British	Third sector organisation	Yes
14	16–25	Male	South Asian/South Asian British	University research volunteer list	Yes

## Data Availability

The data presented in this study are available on request from the corresponding author.

## Supplementary Materials

The following supporting information can be downloaded at: https://www.mdpi.com/article/10.3390/ijerph22111638/s1, Table S1: Supporting extracts for each theme; Supplementary File S2—Interview guide.
