# Supplementary material for: Factors Influencing Online Mental Health Forum Use for People from Ethnic Minority Backgrounds in the United Kingdom: A Mixed Methods Study
Source: Int J Environ Res Public Health. 2025 Oct 28;22(11):1638. doi: 10.3390/ijerph22111638 (PMC12652167; doi:10.3390/ijerph22111638)
Supplement: Supplementary file 1 [file ijerph-22-01638-s001.zip › ijerph-3746200-supplementary.pdf]

Supplementary File S1

Table S1. Supporting extracts for each theme.

Theme	Example quotes	Participant number
1. Sense of community in the online world and offline worlds	*You've got the positive part as well. You've got a little community there that you can lean on and support each other*.	4
	I was on there and I'm still on there and we're still talking even though my baby is [older] but I made some friends there and we still talk.	3
	I guess the thing with [Forum name] as well predominantly cis white males who use [Forum name] so some of the stuff on [Forum name] isn't the best to be looking at especially if you're having mental health crisis episodes.	7
	And local communities and one another yeah. We're much better at reaching out to each other.	10
	Family was harder to get them to understand me, whereas [Forum name] was more… you don't need to go into a big explanation or anything… they have experience with something so they know…	6
	Well, I think like especially east Asian cultures are very polite: to this like if – I'm not saying this doesn't happen in other cultures, I'm sure it does. If it does I haven't seen it but like especially in east Asian cultures if one person has a problem the whole family looks bad. Like if your child has a mental illness so it's a reflection of the family so that's why you have like in a British family but it's particularly pronounced in Chinese families and perfection and high standards.	9
2. Trust is crucial	*…but straight away I listened to my GP because you've got a relationship and a trust with a GP and I really felt very satisfied with my GP surgery and I just listened to him straight away.*	1
	You probably do know this but a lot of stuff to do with mental health in South Asian communities is also to do with religion so I think if you could involve it somehow that like I said that the mosque maybe or something, if you could involve it with religion because a lot of South Asians go to mosque every day – every Friday etcetera so I think involving it somewhere in there would get a lot of people on to it.	7
	It's just I've missed talking about older generations so because back home they might have just seen a doctor all the time so even if it was a junior doctor they would just think, 'Oh it's a doctor,' so doctor is a very trusted label.	2
	I think that it's the same problem as any customer service, I think it needs to be built with trust and without that foundation people will be hesitant to begin with unless their advice is relevant.	5

	Obviously there's always good and bad things but being online you might not trust what people you do not know unless you met them	1
	I think there's a slightly different nuance to that in Asian cultures so my questions are around those sorts of things and it's particularly helpful to get an understanding of the experiences of an Asian person going through that because it is slightly different I think.	9
	Yeah I think ultimately it has to make people feel safe and the fact that what they can confide in isn't necessarily going to be leaked out or gossiped around and what not because they are personal sets of issues so I suppose a lot of it is yeah people feeling like they are safe to speak to one another.	5
	There are some people who have the knowledge that are part of the group and they share credible sources and research and journal articles, so I feel it's a safe space for me to be a part of.	2
	Because it's kind of like feels more safe to do it there and it feels bit like you don't really have to say it out loud, you can just talk about it with someone and they won't judge you and they won't really say anything negative about it.	14
Barriers to accessing online forums	*Technology is already very difficult. Well, I think it is sometimes you know my age group I would say would find it a little bit difficult to use maybe, not very easy but younger generation might find it more appealing to go online, use it, share. I need that human connection. I like that part so for me, it has to be really good for me to go and use it kind of thing. It's similar – like a human connection, so a moderator is there you know to talk to you. Someone is always available kind of thing which I don't know if it can be but younger generations whole world revolves around being online so for them it might be easier to use online forums.*	2
	Sometimes people, you know the elder generation… the elderly I should say, they don't really know about technology	4
	It just feels easy, accessible and it's free, there's no waiting list or waiting time for it	6

Supplementary File S2 – Interview guide

Topic 1: Forum awareness

- Can you tell me where you heard about online mental health forums?
  - What do you understand an online forum to be?
- [*If there is some confusion in the participants understanding of an online forum, then explain what an online forum is*]

Topic 2: Forum use

- Have you ever used a non-mental health online forum?
  - As an observer or poster?
  - Do you know of anyone else who uses these?
- Have you ever used or considered using an online mental health forum?
  - As an observer or poster?
  - Do you know of anyone else who uses these?

Topic 3: Barriers to use

- [*If participant has previously used forum*] Can you tell me things you did not like about online mental health forums?
  - [*If participant indicated a difference in use of mental health and non-mental health forums, explore this difference*]
- [*If participant has not previously used forum*] Can you tell me why you chose not to use an online mental health forum?
  - Any barriers that you think are more significant for people from ethnic minority groups?
- Can you think of ways online forums might not be desirable for others, or might be damaging for others?
  - Specifically for ethnic minority groups

Topic 4: Facilitators of use

- Can you tell me about things you like about [*using, or the idea of using*] online forums?
- Can you think of ways that online mental health forums might help others?
  - Specifically for ethnic minority groups
- Can you explain what would make you more likely to use online mental health forums?
  - Specifically for ethnic minority groups
